# A Case with Significant Proteinuria Caused by Secreted Protein from Urothelial Carcinoma

**DOI:** 10.1155/2011/373480

**Published:** 2011-12-15

**Authors:** Masanori Sakakima, Yoshihide Fujigaki, Hideo Yasuda, Akashi Togawa, Tomoyuki Fujikura, Atsushi Otsuka, Seiichiro Ozono, Akira Hishida

**Affiliations:** ^1^The First Department of Medicine, Hamamatsu University School of Medicine, Higashi-ku, Hamamatsu 431-3192, Japan; ^2^Department of Urology, Hamamatsu University School of Medicine, Hamamatsu, Japan

## Abstract

58-year-old female was admitted to our hospital complaining isolated proteinuria of 1.7 g/day. Abdominal echography showed right-sided unilateral hydronephrosis, and computed tomography pointed out a tumor of the right renal pelvis, suggesting cancer of renal pelvis. The right nephroureterectomy was carried out. Pathological diagnosis was urothelial carcinoma. Renal tissue revealed no apparent glomerulopathy with tubular atrophy, interstitial fibrosis, and mildly-to-moderately interstitial mononuclear cell infiltration. Immunofluorescence study showed no deposition of immunoreactanct, and electron microscopy showed almost normal glomerulus without electron dense deposit. Proteinuria disappeared within 6 days after the operation. Moderate amount of proteinuria in our patient was probably caused by secreted protein from urothelial carcinoma. This condition is rare but should be taken into account in patients with even moderate amount of proteinuria.

## 1. Introduction

It is well known that malignancy causes proteinuria with or without nephrotic syndrome [1–3]. One of the major mechanisms of proteinuria in patients with malignancy is deposition of tumor-related antigen and antibody immune complexes in glomerulus [4]. Urothelial carcinoma may also cause proteinuria by glomerulopathy [5]. Interestingly, secreted protein from urothelial cells into urinary tract can be detected as significant proteinuria [6–8]. However, an amount of proteinuria by secreted protein from carcinoma was reported to be less than 1 g/day [3, 8].

Here we report a patient with moderate amount of proteinuria probably caused by secreted protein of urothelial carcinoma.

## 2. Case Report

58-year-old female was admitted to our hospital because of isolated proteinuria. She was pointed out hypertension and no proteinuria at health checkup in 2005. She suffered from gross hematuria from 1st September, 2007 for one week. She visited F clinic because of proteinuria at health checkup on 28th September, 2007. Urinalysis revealed proteinuria (2+) and occult blood (±). Urinary excretion of protein was 1.7 g/day. Urinary cytology showed class I. She was referred, then admitted to our hospital to evaluate proteinuria on 28th January, 2008.

Physical examination on admission revealed blood pressure of 148/96 mmHg, regular pulse rate of 64 beats/min, and no systemic edema. Laboratory tests showed the following results: blood urea nitrogen 16.5 mg/dL, serum creatinine 0.91 mg/dL, total protein 7.6 g/dL, serum albumin 4.6 g/dL, total cholesterol 242 mg/dL, hemoglobin 13.4 g/dL, white blood cell count 3700/*μ*L, and platelets count 203000/*μ*L. Serological test showed C-reactive protein 0.03 mg/dL, negative for hepatitis B surface antigen, negative for hepatitis C virus antibody, C3 114 mg/dL, C4 104 mg/dL, CH50 60 U/mL, and antinuclear antibody ×40. Urinalysis showed proteinuria of 1.35 g/day without hematuria and cylinduria but with sediments of many transitional cells per high-power field and 0 to 3 leukocytes per high-power field. Urinary chemistry was as follows: N-acetyl-*β*-D-glucosaminidase 6.5 U/L and *β*2-microglobulin 237 *μ*g/L. Creatinine clearance was 103.0 L/day. Urinary cytology showed class V, suggesting urothelial carcinoma.

**Figure 1:**
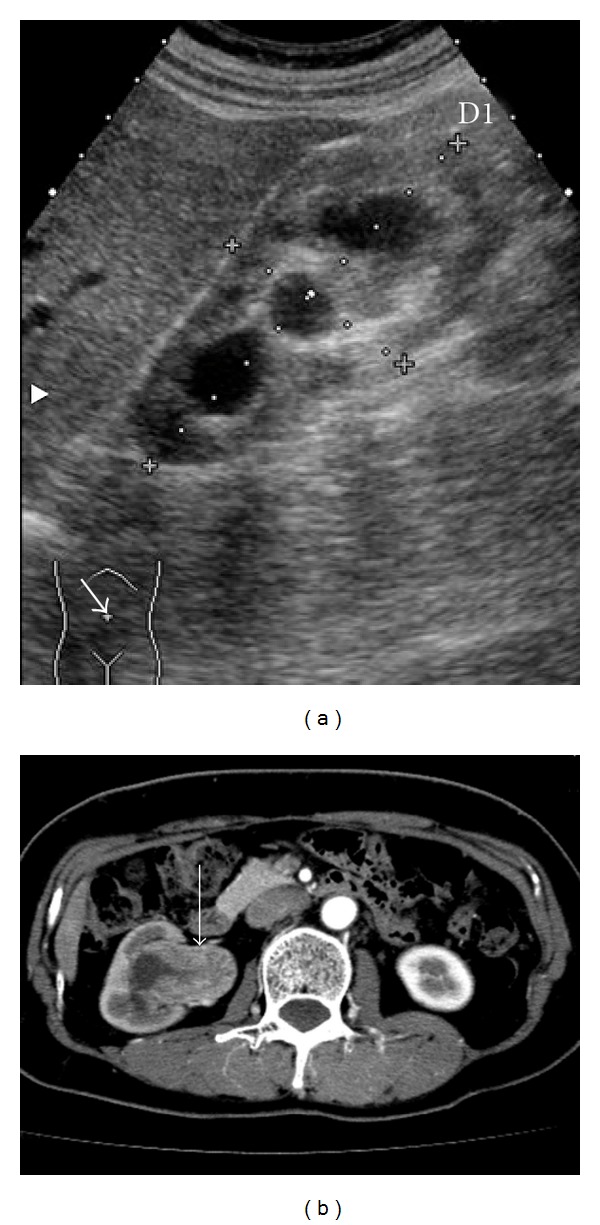
(a) Abdominal echography, showing right-sided unilateral hydronephrosis. (b) Abdominal computed tomography with contrast, showing an enhanced mass in the right renal pelvis (arrow) in association with pelvic dilatation and slight thinning of renal cortex.

**Figure 2:**
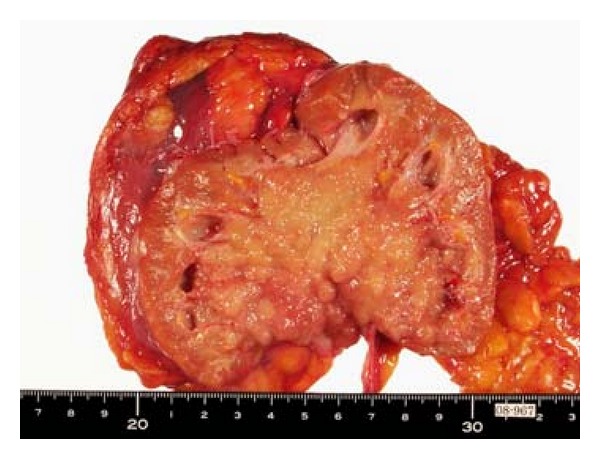
Crosssection photograph of the removed right kidney specimen, showing a tumor of 9 × 6 cm in size in renal pelvis.

**Figure 3:**
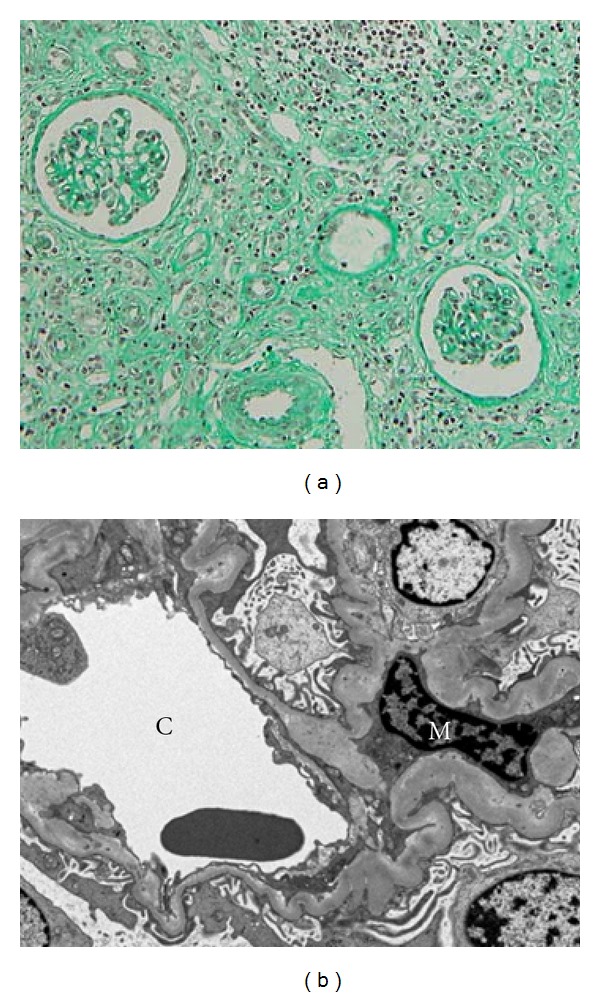
(a) Light microscopic photography, showing slightly ischemic change of glomerulus, tubular atrophy, mononuclear cell infiltration, and interstitial fibrosis (mathon-trichrome staining, ×200). (b) Electron micrograph of glomerulus, showing almost intact morphology without electron dense deposit. C: capillary lumen, M: mesangium.

Chest X-ray examination was found to be normal. Abdominal echography showed right-sided unilateral hydronephrosis (Figure 1(a)), and abdominal computed tomography with contrast media pointed out a mass in the right renal pelvis in association with pelvic dilatation and slight thinning of renal cortex (Figure 1(b)), strongly suggesting cancer of renal pelvis. The right kidney was totally nephroureterectomized (Figure 2) on 29th February, 2008. Pathological diagnosis was urothelial carcinoma of renal pelvis (G2, INF*β*, pT1, rt-u0, ew0, ly0, v0) (data not shown). Renal histological examination revealed slightly ischemic change of glomeruli without apparent glomerulopathy in association with tubular atrophy and interstitial fibrosis with mildly-to-moderately mononuclear cell infiltration in 60% of cortical areas (Figure 3(a)). Immunofluorescence study showed no deposition of immunoreactanct in the renal tissue and electron microscopy showed almost intact glomerulus without electron dense deposits (Figure 3(b)).

**Figure 4:**
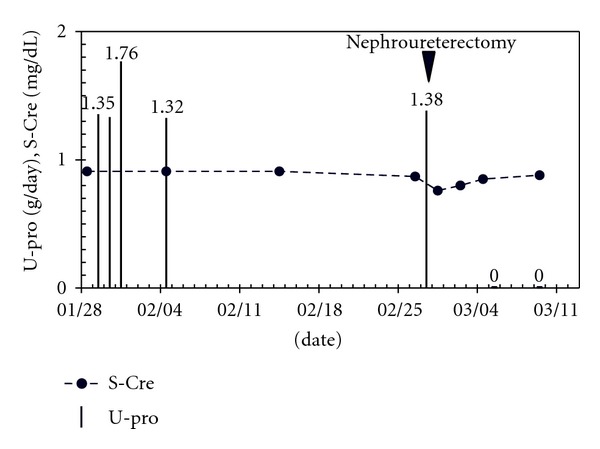
Clinical course of the patient.

The patient discharged because that proteinuria disappeared within 6 days after the operation (Figure 4).

## 3. Discussion

It is well known that malignancy sometimes associates nephrotic syndrome [1, 2]. Malignancy-associated glomerular diseases may present most types of glomerulopathy [9]. Among them, membranous nephropathy by tumor-related antigen-antibody immune complexes in solid tumors is most commonly encountered [1]. Malignancy is also associated with nonnephrotic level proteinuria, however; cause of proteinuria is not clear because that histological examination of them is rare [3].

Hemmingsen et al. analyzed urinary protein profiles in patients with extrarenal epithelial carcinoma [3], renal carcinoma [10], and transitional cell carcinoma of the bladder [5] and reported that carcinoma-associated proteinuria consisted of much higher amount of high molecular weight protein such as albumin, transferring, haptoglobin, IgG, IgA, and IgM when compared with apparently healthy subject, suggesting that proteinuria is caused by glomerular injury in carcinoma-associated proteinuria. Moreover, Hemmingsen et al. reported that an amount of protein excretion in urine was not decreased in some patients with transitional cell carcinoma of the bladder after urinary diversion [5] and that an amount of urinary protein may be correlated with concentration of circulating immune complexes [11], indicating that immune complex-mediated glomerulopathy should cause proteinuria in some patients with transitional cell carcinoma of the bladder.

 However, there were a few reports that performed renal histological examinations. It is reported that a patient with transitional cell carcinoma of the bladder showed immune complex formation in nephrectomized kidney but no proteinuria [12]. A patient with transitional cell carcinoma of the bladder presenting nephrotic syndrome showed minor glomerular abnormalities with linear IgG deposition in glomerular capillary walls by immunofluorescence study [13]. They proved that antibody formation against a specific component of basement membrane, common to both kidney and tumor, gave rise to the nephropathy in this case. It took about 6 weeks that proteinuria disappeared after the tumor removal in this case.

On the other hand, Johansson reported that secreted protein from urothelial carcinoma may result in significant proteinuria [6–8]. They reported that concentration of urinary IgM in urinary carcinomas such as pelvic carcinoma was significantly much more when compared with that in glomerulonephritis [6]. They examined immunohistochemistry of IgM between carcinoma lesion and normal tissue from resected urinary bladder and found that interstitial cells in the carcinoma contained significant amount of IgM [7]. Patients with IgM-positive tumor tissue were found to excrete more IgM into urine than patients with IgM-negative tumor tissue, concluding that secreted IgM from carcinomas may contribute to urinary IgM [7]. Again, they also examined the relationship among tumor size, disease stage, degree of malignancy, and amount of urinary proteins such as albumin, transferring, IgG, IgA, and IgM in 59 patients with urothelial tumors and found significant relation between the amount of urinary protein and tumor size, suggesting that proteinuria should be derived from secreted protein of tumors [8]. Zhau et al. reported 180 kda protein in urine of 78 out of 105 patients (74.3%) with transitional cancer of the urinary bladder by using sodium dodecyl sulfate polyacrylamide gel electrophoresis and found that tumor homogenate not normal tissue homogenate included the protein [14], also indicating that the protein was secreted from the tumor into urine [6–8]. An amount of urinary protein excretion was mostly reported to be less than 1 g/day [3, 8].

 Our case showed tubulointerstitial damages without significant increase in tubular protein and urinary NAG and no apparent glomerulopathy, suggesting that it is less likely that proteinuria was either tubular or glomerular origins. Moreover, proteinuria persisted constantly just before her operation, however, proteinuria of about 1.5 g/day completely disappeared within at most 6 days after the right nephroureterectomy. Afzali et al. [15] reported a pregnant case where nephrotic syndrome was associated with gross unilateral ureteric obstruction and protein leak was documented from both the obstructed, and unobstructed kidneys. Protein leak did not resolve after delivery, but remission needed a laparoscopic pyeloplasty. Our case had also unilateral hydronephrosis, but it was mild, and the right nephroureterectomy soon brought disappearance of proteinuria, suggesting that the cause of proteinuria may be different from the case by Afzali et al. [15] Although urinary protein profile was not analyzed in our patient, taken together, it is likely that proteinuria in our patient may be derived from secreted protein from urothelial carcinoma. 2+ positive albumin dipstick urinalysis may suggest that albumin predominates as the cause of proteinuria in our case.

This condition is very rare but should be taken into account in patients with even moderate proteinuria.

## References

[1] Eagen JW, Lewis EJ (1977). Glomerulopathies of neoplasia. *Kidney International*.

[2] Lee JC, Yamauchi H, Hopper J (1966). The association of cancer and the nephrotic syndrome. *Annals of Internal Medicine*.

[3] Hemmingsen L, Skaarup P (1977). Urinary excretion of ten plasma proteins in patients with extrarenal epithelial carcinoma. *Acta Chirurgica Scandinavica*.

[4] Couser WG, Salant DJ (1980). In situ immune complex formation and glomerular injury. *Kidney International*.

[5] Hemmingsen L, Rasmussen F, Skaarup P, Wolf H (1981). Urinary protein profiles in patients with urothelial bladder tumours. *British Journal of Urology*.

[6] Johansson B, Kistner S, Norberg R (1971). Proteinuria in patients with urinary tract tumours. *Scandinavian Journal of Urology and Nephrology*.

[7] Johansson B, Ljungqvist A (1974). Localization of immunoglobulins in urinary bladder tumours. *Acta Pathologica Microbiologica Scandinavica A*.

[8] Johansson B, Kistner S (1975). Proteinuria in patients with uroepithelial tumours with special regard to tumour size, clinical staging and grade of malignancy. *Scandinavian Journal of Urology and Nephrology*.

[9] Davison AM, Hartley B, Davison AM, Cameron JS, Grundeld JP (2005). Malignancy-associated glomerular disease. *Oxford Textbook of Clinical Nephrology*.

[10] Hemmingsen L, Skaarup P (1977). Urinary excretion of ten plasma proteins in patients with renal carcinoma. *Scandinavian Journal of Urology and Nephrology*.

[11] Skaarup P, Jensenius JC, Brandslund I, Svehag SE, Wolf H (1984). Association of circulating immune complexes with glomerular proteinuria in patients with transitional cell carcinoma of the urinary bladder. *European Urology*.

[12] Jones LW, Levin A, Fudenberg HH (1975). Glomerular antigen complexes associated with transitional cell carcinoma. *Surgery Gynecology and Obstetrics*.

[13] Rapoport J, Kuperman O, Gopas Y (1989). Nephrotic syndrome associated with transitional cell carcinoma of the bladder. *Nephron*.

[14] Zhau HE, Babaian RJ, Hong SJ (1990). A new 180 kDa. urine protein marker associated with bladder cancer. *Journal of Urology*.

[15] Afzali B, Kingdon E, Holt SG (2005). Treatment of unilateral obstruction reversing heavy and bilateral proteinuria. *Nephrology Dialysis Transplantation*.

